# Structural profile of dome-shaped macula in degenerative myopia and its association with macular disorders

**DOI:** 10.1186/s12886-020-01473-2

**Published:** 2020-05-24

**Authors:** Geun Woo Lee, Jae Hui Kim, Se Woong Kang, Jaeryung Kim

**Affiliations:** 1 Department of Ophthalmology, Samsung Medical Center, Sungkyunkwan University School of Medicine, #81 Irwon-ro, Gangnam-gu, Seoul, 06351 South Korea; 2 Department of Ophthalmology, Kim’s Eye Hospital, Konyang University College of Medicine, #131 Yeounsin-ro, Yeongdeungpo-gu, Seoul, South Korea

**Keywords:** Dome-shaped macula, Myopic foveoschisis, Degenerative myopia

## Abstract

**Background:**

To evaluate the detailed structural profile of dome-shaped macula and its association with myopic macular complications.

**Methods:**

This retrospective study included 147 eyes of 93 patients who were diagnosed with degenerative myopia. The height of the scleral dome and diameter of the dome base were measured via enhanced depth imaging optical coherence tomography images with 1:1 μm setting. Spherical equivalent and best-corrected visual acuity were compared in eyes with and without dome-shaped macula. In eyes with dome-shaped macula, the height and diameter of the dome were compared in eyes with and without myopic macular complications including choroidal neovascularization, myopic foveoschisis, and macular hole.

**Results:**

Dome-shaped macula was noted in 60 eyes (40.8%) of 42 patients. The mean height of the dome in the eyes with dome-shaped macula was 126.5 ± 69.4 μm (53 to 345 μm) and the mean diameter of the dome base was 2862.1 ± 794.9 μm (1567 μm to 4886 μm). In comparing eyes with and without dome-shaped macula, eyes with dome-shaped macula had higher myopia (− 13.7 diopters vs − 12.1 diopters, *P* = 0.022). There was no difference in visual acuity in eyes with or without dome-shaped macula (*P* = 0.132). The height and diameter of the dome in eyes with and without myopic foveoschisis were 78.6 ± 20.6 μm and 134.9 ± 71.6 μm, 2499.2 ± 303.1 μm and 2969.3 ± 645.7 μm, respectively (*P* = 0.009 and *P* = 0.017). However, the height and diameter of the dome were not related to the incidence of a macular hole (*P* = 0.324 and *P* = 0.605) and choroidal neovascularization (*P* = 0.835 and *P* = 0.905).

**Conclusions:**

The prevalence of dome-shaped macula was about 40% in the eyes with degenerative myopia. Although dome-shaped macula was associated with higher degrees of myopia, a prominent dome seemed to be protective against myopic foveoschisis.

## Background

The dome-shaped macula is a distinct entity characterized by convex elevation of the macula in high myopia [1]. This elevation of the macula is often negligible on fundus examination or ultrasonography, but can be clearly detected via an optical coherence tomography (OCT) examination [1]. Enhanced depth imaging (EDI) OCT examination revealed that the elevation is the result of a relatively thick sclera under the macula [2]. In addition, eyes with dome-shaped macula exhibited thicker choroid than eyes those without [2].

Although tomographic features of dome-shaped macula have been investigated [2, 3], paucity of knowledge exists regarding the clinical significance of this peculiar entity. Several investigators suspected possible association of dome-shaped macula with foveal detachment [1, 4] and choroidal neovascularization [2, 3]. One previous study suggested that macular elevation may prevent retinoschisis and retinal detachment related to macular hole [5]. A more recent study by Liang et al. suggested that a dome-shaped macula was associated with an increased incidence of foveal detachment and a decreased incidence of myopic foveoschisis [6]. However, potential associations between ocular disorders and dome height have not yet been fully elucidated.

EDI-OCT is a technique that facilitates better visualization of deep ocular tissue, including choroid and sclera [2, 7, 8]. The purpose of the present study was to investigate the detailed structural profile of the dome-shaped macula using EDI-OCT and to evaluate its association with myopic macular disorders. We focused on the association between dome height and such disorders.

## Methods

This retrospective observational study was performed at a single center according to the tenets of the Declaration of Helsinki. The study was prospectively approved by the Institutional Review Board of the Samsung Medical Center.

We retrospectively reviewed the medical records of patients with degenerative myopia. A computerized search was performed using the term “myopi-” and “degen-“in records of patients who were examined at the vitreo-retinal clinic of the Samsung Medical Center.

To be included in this study, all subjects were required to have undergone a comprehensive ophthalmologic examination that included measurements of best-corrected visual acuity (BCVA), manifest refraction, slit-lamp biomicroscopy, and fundus examination. In eyes that had undergone cataract surgery or refractive surgery, refractive error before the surgery was taken for analysis. Only eyes with greater than − 6.0 diopters of myopia were included. The BCVAs were transformed to a logMAR (minimal angle of resolution) scale for analysis. All patients had also had fundus photographs and EDI-OCT examination.

The horizontal and vertical EDI-OCT crosshair scans centered at the center of the fovea were routinely conducted in those eyes using spectral domain OCT (Spectralis, Heidelberg Engineering GmbH, Heidelberg, Germany). The EDI image was obtained either via the conservative method by pushing the instrument close to the eye [8] until year 2010 or via Spectralis enhanced depth imaging mode by pressing the conversion button provided in the Spectralis software afterwards. To improve visualization, the values of 50 to 100 scans were averaged for each section.

The scleral thickness and choroidal thickness was measured manually for all the included eyes via the Heidelberg Eye Explorer software (version 1.7.0.0). Since a previous study demonstrated the possible overestimation of tissue thickness in measurements based on 1:1 pixel images [9], all the measurements were performed through 1:1 μm images. Scleral thickness was defined as the distance between the inner and outer border of the sclera. The measurement line was drawn as a perpendicular line between the two borders. The mean value from horizontal and vertical scans was used for analysis. Choroidal thickness was defined as the distance from the hyperreflective line of the Bruch’s membrane to the chorio-scleral interface. The measurement line was also drawn as a perpendicular line between Bruch’s membrane and the chorio-scleral interface. The subfoveal choroidal thickness was measured based on both horizontal and vertical scans. The measurements were also performed at 500 μm in the superior, inferior, nasal, and temporal directions from the foveal center. The average choroidal thickness of all six measurements was defined as the central choroidal thickness. When the choroidal thickness was immeasurable due to severe choroidal thinning or atrophy, the thickness was assumed to be 1 μm.

**Fig. 1:**
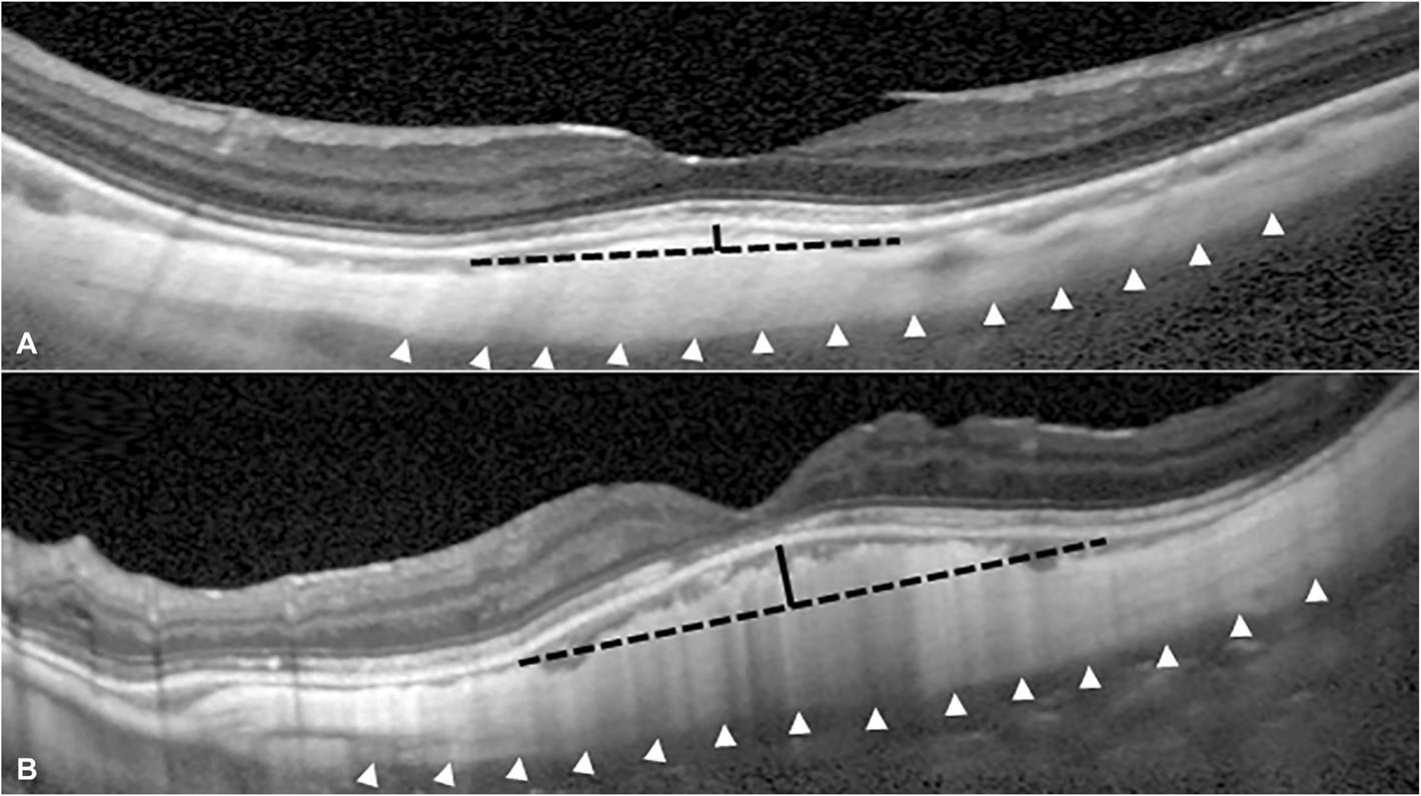
Representative vertical optical coherence tomography of the 1:1 μm setting of an eye with a dome-shaped macula. **a** Dome-shaped macula without inferior staphyloma (dome height = 85 μm, dome diameter = 2167 μm, subfoveal scleral thickness = 442 μm). **b** Dome-shaped macula with inferior staphyloma (dome height = 234 μm, dome diameter = 2716 μm, subfoveal scleral thickness = 561 μm). Both images have a short black line (measurement line for dome height) and a long black dotted line (measurement line for dome diameter). The outer scleral border is marked by white arrowheads. Note obvious subfoveal scleral thickening in the eye with inferior staphyloma

The confirmative diagnosis of dome-shaped macula was made based on the result of height measurement. The height and diameter of the dome region were measured for eyes with dome-shaped macula. A line connecting the border of the dome-shaped sclera was drawn and the length of the line was defined as the diameter of the dome base (Fig. 1). Another perpendicular line which starts at the top of the dome and ends at the measurement line for the diameter of the dome base was drawn. The length of this second line was defined as the height of the dome (Fig. 1). The maximum height of the dome among the measurements based on horizontal and vertical scans was used for analysis. Two different cut-off values were used. Based on previous experience, we could identify the definite shape of dome-shaped macula on OCT when the height of dome exceeds approximately 50 μm. Thus, we determined to diagnose dome-shaped macula when more than 50 μm of the height of the dome was noted in either a horizontal or a vertical scan. A similar cut-off value was used in the previous studies [3, 10]. To evaluate possible differences in results due to cut-off values, 150 μm was set as another cut-off value. Both cut-off values were used to compare the differences in incidence of macular disorders between eyes with and without dome-shaped macula. All the measurements were performed with the verification of two examiners (J.H.K. and J.K.). Additional analysis was performed to evaluate whether the 50 μm cut-off value is appropriate to diagnose dome-shaped macula. To perform analysis, two examiners blinded to the height of the dome independently and intuitively evaluated the presence of dome-shaped macula based on OCT images. The presence of a dome-shaped macula was confirmed when the two examiners both noted the feature. The receiver operating characteristic curve for dome height was obtained, and the optimal cut-off value was determined using Youden’s index. After that, the optimal cut-off value was compared with 50 μm.

The association of the height of the dome with the diameter of the dome, spherical equivalent, and BCVA was analyzed. The association of the diameter of the dome with the spherical equivalent and BCVA was additionally analyzed. The incidence of macular disorders, including choroidal neovascularization, myopic foveoschisis, and macular hole was compared in eyes with dome-shaped macula and eyes without dome-shaped macula. The diagnosis of myopic foveoschisis was made when retinoschisis involved the fovea. Subgroup analysis was performed for eyes with dome-shaped macula. In eyes with dome-shaped macula, the height of the dome and the diameter were compared between eyes with and without choroidal neovascularization, myopic foveoschisis, and macular hole.

Based on the findings in the fundus photograph and vertical OCT scan, eyes were divided into dome-shaped macula with inferior staphyloma and dome-shaped macula without inferior staphyloma (Fig. 1). The presence of inferior staphyloma was diagnosed when the steep slope of the retinal/choroidal image plane in the inferior direction was observed in the vertical section of the OCT image. The fundus photographs were used as adjunctive data to identify findings known to be associated with inferior staphyloma, such as inferonasal tilting of the disc, and degenerative changes evident in the inferior macula. The distribution of eyes within the two groups was compared in eyes with dome-shaped macula and eyes without dome-shaped macula. In eyes with dome-shaped macula, the subfoveal scleral thickness was additionally compared between eyes with and without inferior staphyloma.

Statistical analyses were performed with the commercially available software package (SPSS ver. 18.0 for Windows; SPSS Inc., Chicago, IL, USA). Comparisons of scleral thickness and choroidal thickness were performed using independent samples *t* test. Prevalence of macular disorders between eyes with and without dome-shaped macula was compared using chi-square test or Fisher’s exact test. The associations of the height and the diameter of the dome with the spherical equivalent and BCVA were analyzed using Pearson’s correlation analysis. Comparisons of the height of the dome in eyes with and without macular disorders were performed using a Mann–Whitney *U* test. The distribution of eyes in the staphyloma group was compared using a Chi-square test. A *P*-value less than 0.05 was considered significant.

## Results

The database included a total of 144 patients. Among them, 154 eyes of 99 patients met the inclusion criteria. The other patients were excluded for the following reasons: the refractive error was lesser than − 6.0 D, preoperative refractive error could not be verified in cases with a history of cataract surgery or refractive surgery, or the EDI-OCT image was not available. Of those patients, eight eyes of six patients were additionally excluded because the scleral thickness could not be accurately measurable due to a relatively thick choroid. Eventually, data of 147 eyes of 93 patients were included in the result analysis.

**Fig. 2:**
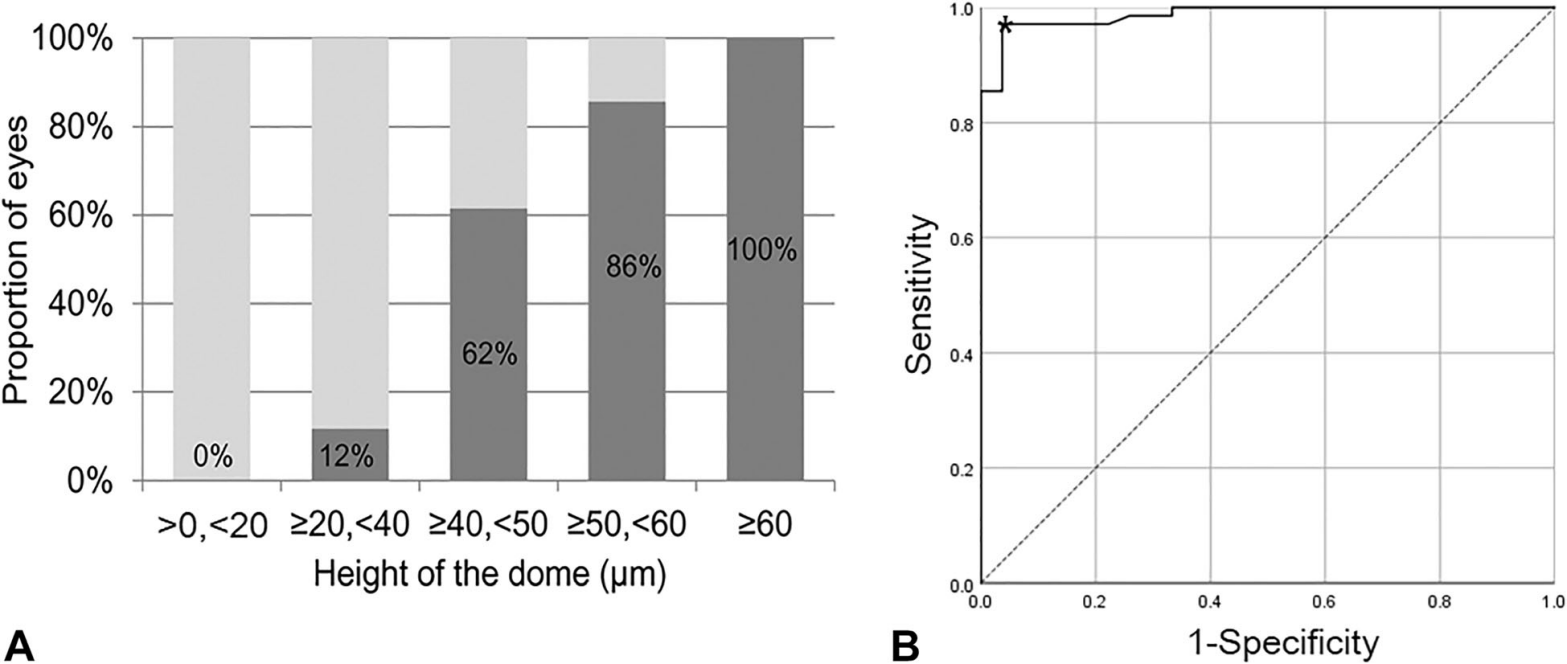
**a** Among the 97 eyes with a measurable dome, the bar graphs show the dome height distribution in the 70 eyes first diagnosed with the dome-shaped macula. **b** The receiver operating curve was used to obtain the optimal cut-off value (asterisk [*] = 48.5 μm)

The number of eyes with a dome with measurable height was 97. Among these, 70 eyes were intuitively diagnosed as having a dome-shaped macula on OCT. The distribution of the height of the dome in 70 eyes is shown in Fig. 2a. The optimal cut-off value was 48.5 μm, which was determined using the receiver operating characteristic curve and Youden’s index (Fig. 2b, area under the curve = 0.988, sensitivity = 0.971, specificity = 0.963). Through agreement analysis, 48.5 um was found to have almost perfect agreement when compared to the 50-um cut-off value we first used (Cohen’s kappa coefficient: 0.933, *P* < 0.001). A confirmative diagnosis of dome-shaped macula with a dome height over 50 μm was made in 60 eyes (40.8%) of 42 patients (24 horizontal scans and 55 vertical scans) with degenerative myopia.

Eighteen patients exhibited bilateral dome-shaped macula. The mean age of the 42 patients was 55.1 ± 13.0 years (mean ± standard deviation). In eyes with dome-shaped macula, the mean spherical equivalent was − 13.7 ± 4.8 and the mean BCVA was 0.54 ± 0.42. Subfoveal scleral thickness and central choroidal thickness was 401.2 ± 98.9 μm and 37.6 ± 21.4 μm, respectively.

Myopic choroidal neovascularization, myopic foveoschisis, and macular holes were noted in 10 eyes (16.7%), 9 eyes (15.0%), and 6 eyes (10.0%), respectively. Retinal detachment related to a macular hole was observed in one of the 6 eyes with a macular hole. The height of the dome in that eye was 132 μm. Among eyes with dome-shaped macula without other myopic macular complications (e.g., choroidal neovascularization, myopic foveoschisis, or macular hole), two of 35 eyes (5.7%) had macular serous detachment (i.e., foveal detachment). Table 1 compares characteristics of eyes with dome-shaped macula and eyes without dome-shaped macula.

**Table 1:** Comparisons of characteristics of the eyes with and without dome-shaped macula

Characteristic	Eyes with dome-shaped macula (*N* = 60)	Eyes without dome-shaped macula (*N* = 87)	*P*-value
Spherical equivalent, diopters	−13.7 ± 4.8	−12.1 ± 3.9	0.022*
Best-corrected visual acuity	0.54 ± 0.42	0.43 ± 0.45	0.132*
Subfoveal scleral thickness, μm	401.3 ± 98.9	315.7 ± 93.5	< 0.001*
Central choroidal thickness,μm	37.6 ± 21.4	44.6 ± 27.3	0.082*
Macular complications, n (%)			
Choroidal neovascularization	10 (16.7%)	20 (22.9%)	0.409^†^
Myopic foveoschisis	9 (15.0%)	16 (18.4%)	0.660^†^
Macular hole	6 (10.0%)	2 (2.3%)	0.063^††^

* Statistical significance was tested using independent samples *t* test

^†^Statistical significance was tested using chi-square test

^††^Statistical significance was tested using Fisher’s exact test

Dome-shaped macula was not noted in 87 eyes (59.2%) of 64 patients, and the mean age of patients was 58.1 ± 11.4 years. In eyes without dome-shaped macula, the mean spherical equivalent was − 12.1 ± 3.9 and the mean BCVA was 0.43 ± 0.45. Subfoveal sclera thickness and central choroidal thickness were 315.7 ± 93.5 μm and 44.6 ± 27.3 μm, respectively. Choroidal neovascularization, myopic foveoschisis, and macular holes were noted in 20 eyes (22.9%), 16 eyes (18.4%), and 2 eyes (2.3%), respectively. Although eyes with dome-shaped macula had a greater degree of myopia than the eyes without dome-shaped macula (*P* = 0.022), BCVA was not different between the two groups (*P* = 0.132). BCVA was not different between the two groups when analyzed in eyes without macular disorders (*P* = 0.692). The incidence of choroidal neovascularization, myopic foveoschisis, and macular holes was not different between the two groups (*P* = 0.409, *P* = 0.660, and *P* = 0.063, respectively). The subfoveal sclera was significantly thicker in eyes with dome-shaped macula (*P* < 0.001). However, difference in central choroidal thickness between the two groups was not significant (*P* = 0.082).

In comparison, using 150 μm as a cut-off value, 17 eyes were diagnosed with dome-shaped macula. Among them, two eyes (11.8%) were found to have choroidal neovascularization and two eyes (11.8%) were found to have a macular hole. Myopic foveoschisis was not observed. The incidences of choroidal neovascularization (*P* = 0.525) and macular hole (*P* = 0.232) were not different between eyes with (*n* = 17) and without (*n* = 104) dome-shaped macula. Although eyes with dome-shaped macula exhibited a relatively lower incidence of myopic foveoschisis than eyes without it, the difference was not significant (*P* = 0.078).

In the eyes with dome-shaped macula defined using a cut-off value of 50 μm, the mean height of the dome was 126.5 ± 69.4 μm (53 μm to 345 μm) and the mean diameter of the dome base was 2862.1 ± 794.9 μm (1567 μm to 4886 μm). There was a significant positive correlation between the height of the dome and the diameter of the dome base (*P* < 0.001, r = 0.470). Also, the height of the dome was positively correlated with subfoveal scleral thickness (*P* = 0.003, r = 0.378). However, the height of the dome was not correlated with BCVA or spherical equivalent (*P* = 0.489 and *P* = 0.479, respectively). Also the diameter of the dome base was not correlated with BCVA and spherical equivalent (*P* = 0.096, and *P* = 0.285, respectively). The association of the height of the dome and the diameter of the dome base with BCVA and spherical equivalent were not significant when analyzed in eyes without macular disorders (*P* > 0.05).

In the analysis of the eyes with dome-shaped macula, the heights of the dome in eyes with and without choroidal neovascularization were 127.7 ± 70.5 μm and 126.2 ± 69.9 μm, respectively. The values were 78.6 ± 20.6 μm and 134.9 ± 71.6 μm in eyes with and without myopic foveoschisis, respectively, and 164.0 ± 104.6 μm and 122.3 ± 64.4 μm in eyes with and without macular holes, respectively. The height of the dome in eyes without myopic foveoschisis was significantly greater than in eyes with myopic foveoschisis (Table 2, *P* = 0.009). The maximum height of the dome among the nine eyes with myopic foveoschisis was 114 μm. The difference in the height of the dome was not significant in eyes with or without choroidal neovascularization and macular holes (*P* = 0.835 and *P* = 0.324). Again, the difference in the height of the dome was not significant in eyes with or without epiretinal membrane, vitreomacular traction, or inferior staphyloma (Table 2). In addition, the correlation of dome diameter with the presence of complications showed significance only in myopic foveoschisis (Table 2, *P* = 0.017). In eyes with and without dome-shaped macula, inferior staphyloma was noted in 12 eyes (20.0%) and 15 eyes (17.2%), respectively. The incidence of inferior staphyloma was not different in eyes with or without dome-shaped macula (*P* = 0.671), and prominent thickening of subfoveal sclera was noted regardless of the type of staphyloma. In eyes with dome-shaped macula, subfoveal scleral thicknesses in eyes with and without inferior staphyloma were 448.8 ± 94.5 μm and 395.0 ± 98.7 μm, respectively (*P* = 0.209).

**Table 2:** Comparisons of the height of the dome and the diameter of the dome between eyes with and without myopic macular complications, as the subgroup analysis of the eyes with dome-shaped macula

Macular complication	With complication (n)	Without complication (n)	***p***-value*
**Comparisons of the height**			
Choroidal neovascularization	127.7 ± 70.5 μm (10)	126.2 ± 69.9 μm (50)	0.835
Myopic foveoschisis	78.6 ± 20.6 μm (9)	134.9 ± 71.6 μm (51)	0.009
Macular hole	164.0 ± 104.6 μm (6)	122.3 ± 64.4 μm (54)	0.324
Epiretinal membrane	110.8 ± 38.5 μm (10)	129.6 ± 73.9 μm (50)	0.439
Vitreomacular traction	111.9 ± 50.4 μm (11)	129.7 ± 72.9 μm (49)	0.446
Inferior staphyloma	128.3 ± 82.6 μm (12)	126.2 ± 68.3 μm (48)	0.941
**Comparisons of the diameter**			
Choroidal neovascularization	2877.1 ± 419.9 μm (10)	2789.9 ± 696.2 μm (50)	0.905
Myopic foveoschisis	2499.2 ± 303.1 μm (9)	2969.3 ± 645.7 μm (51)	0.017
Macular hole	2920.3 ± 369.7 μm (6)	2896.5 ± 652.8 μm (54)	0.605
Epiretinal membrane	2724.4 ± 494.3 μm (10)	2933.7 ± 650.1 μm (50)	0.415
Vitreomacular traction	2805.7 ± 359.9 μm (11)	2919.8 ± 674.8 μm (49)	0.748
Inferior staphyloma	2843.0 ± 449.3 μm (12)	2912.8 ± 668.5 μm (48)	0.824

*Statistical significance was tested using Mann-Whitney *U* test

## Discussion

This study focused on identifying the association between dome-shaped macula and representative myopic complications. Dome-shaped macula was noted in approximately 40% of the eyes with degenerative myopia. In a manner consistent with the previous studies [1, 5, 6], a dome-shaped macula was more frequently identified in vertical scans than in horizontal scans and the degree of myopia was greater in eyes with a dome-shaped macula than in those without. A definite association between dome-shaped macula and visual acuity was not verified. The subfoveal scleral thickness was significantly greater in eyes with dome-shaped macula than those without dome-shaped macula. However, there was no difference in choroidal thickness. Although the incidence of macular disorders was not different in the two groups, significantly lower incidence of myopic foveoschisis was noted in eyes with a relatively high dome.

The original definition of dome-shaped macula which was established by Gaucher and associates is a “convex elevation of macula.” [1] There is no definite guideline on the exact degree of elevation in diagnosing dome-shaped macula. Coco and associates [5] defined a height greater than 250 μm of the dome as a “clear intrusion,” whereas more than 50 μm of inward bulge of the retinal pigment epithelium in the OCT was defined as dome-shaped macula in the other studies [3, 6]. In the present study, dome-shaped macula was diagnosed when the height of the dome exceeded 50 μm.

The height of the dome was relatively lower than that in previous studies [2, 3, 6]. Possible explanations for this discrepancy are as follows: first, the difference in definition of dome-shaped macula may have an influence. In this study, only a small amount of scleral elevation was considered to be dome-shaped macula. We suspect that the definition of “convex elevation of macula” in previous studies might be stricter than our definition [2]. Second, the possible disparity between measurements based on 1:1 μm images and 1:1 pixel images may also have some influence. A previous study demonstrated the overestimation of tissue thickness via measurement based on horizontally compressed 1:1 pixel images when the measurement line is not vertical [9]. Considering various scleral curvatures of posterior poles in eyes with degenerative myopia [11], straight vertical measurement of the dome height may not be possible in many eyes. Measurements based on a 1:1 μm setting might reflect the real height of the dome more accurately.

In the present study, two different types of dome-shaped macula have been noted. The first type is a classic dome-shaped macula that shows a radially symmetric shape (Fig. 1a). The second type is associated with a variation in the inner scleral contour as a result of inferior staphyloma (Fig. 1b). Maruko and associates evaluated the scleral thickness in eyes with tilted disc syndrome. The superior edge of staphyloma in the eyes included in that study was located at the fovea. In a manner similar to scleral thickening in eyes with a dome-shaped macula, the subfoveal sclera in the included eyes was thicker than that of other areas [12]. The scleral profile in the representative OCT images presented by Maruko et al. was similar to that of second type dome-shaped macula of the present study. Although both types of dome-shaped macula in the current study exhibit a thickening of the subfoveal sclera [2], it is not certain whether these two types of dome-shaped macula share the same etiology. In the current study, the height of the dome and subfoveal scleral thickness was not different between eyes with and without inferior staphyloma. This indicates that the presence of inferior staphyloma may not influence the shape of the dome. The possible difference in the influence of retinal and choroidal profiles between the two types of dome-shaped macula warrants further investigation.

Dome-shaped macula has been associated with visual impairment [1, 13]. In the present study, there was no difference in BCVA between eyes with a dome-shaped macula and eyes without, even in eyes without macular disorders. Also, the association between BCVA and the height of the dome was not significant in eyes with dome-shaped macula.

Previously, Coco and associates postulated that a dome-shaped macula may prevent retinoschisis at the top of the bulge [5]. In a study by Liang et al., the incidence of fovea-involving retinoschisis was significantly lower in eyes with a dome-shaped macula than in eyes without [6]. In the present study, we first investigated whether dome height is associated with retinal disorders. We found that dome height and dome diameter were significantly lower in eyes with myopic foveoschisis. The similarity in this result among these two parameters is likely due to the positive correlation between dome height and dome diameter. It is likely that dome-shaped macula has some valid role in preventing myopic foveoschisis probably by releasing the traction on the fovea when the dome is high enough. The maximum height of the dome among the eyes with myopic foveoschisis was 114 μm. Thus, it is likely a dome height greater than 114 μm may have some preventive effect on the development of myopic foveoschisis. Further studies with a different cohort are warranted to confirm the finding. There were discrepancies between the results of the current study and those of the previous studies regarding the association between dome-shaped macula and other myopic macular complications. In the present study, the mean height of the dome was not different between the eyes with or without choroidal neovascularization. However, in the study by Ellabban and associates, the mean height of the dome was significantly lower in eyes with choroidal neovascularization than in eyes without it [3]. The incidence of choroidal neovascularization was relatively high, and ranging up to about 40%, in the eyes in their study. We suspect that the eyes included in the study and the definition of choroidal neovascularization would be different in the two studies. Coco and associates hypothesized that dome-shaped macula may also prevent retinal detachment related to macular holes based on the result that none of their 3 patients with macular holes developed retinal detachment [5]. However, our finding does not support this postulation. The history of retinal detachment related to macular holes was noted in one eye with 132 μm of height of dome. Dome-shaped macula may have some effect similar to a macular buckling procedure by pushing the retina towards the vitreous cavity. However, the effect of dome-shaped macula is likely limited because all our patients had no more than 350 μm of height of the dome whereas representative OCT images after macular buckling procedure [14, 15] show that the elevation induced by buckling is greater than several times the retinal thickness. A further large-scale study may provide a clear answer for the association of the height of the dome with the incidence of choroidal neovascularization, macular holes, and retinal detachment.

There has been some debate regarding the definition of dome-shaped macula. Beyon and Chu believe that the dome-shaped macula may be a feature of a complicated staphyloma type or a variant of inferior staphyloma and suggested to use the term “sclera compression maculopathy [16].” However, Spaide and Imamura claimed that thickening of the sclera under the fovea is a unique finding distinct from ordinary staphyloma [17]. More recently, Coco and associates classified dome-shaped macula as a posterior staphyloma and inferior staphyloma groups. Posterior staphyloma was noted in 48 of 68 eyes (70.6%) whereas inferior staphyloma was noted in the other 20 eyes (29.4%) [5]. However, the possible difference in scleral structural profile between the two groups was not investigated because they used a previous version of OCT that had limited penetration into the deep tissue. In the present study, the incidence of inferior staphyloma was not different between the eyes with dome-shaped macula and the eyes without dome-shaped macula, and a prominent thickening of the subfoveal sclera was noted regardless of the type of staphyloma. In eyes with dome-shaped macula, we could verify relative thickening of sclera at the subfoveal lesion regardless of the type of staphyloma. The mean subfoveal scleral thickness of eyes without inferior staphyloma was 395 μm, approximately 80 μm thicker than the thickness of eyes without dome-shaped macula. We believe that these findings may support the suggestion made by Spaide and Imamura [17]. However, one interesting finding is that the mean subfoveal scleral thickness of eyes with inferior staphyloma was approximately 54 μm thicker than eyes without inferior staphyloma in eyes with dome-shaped macula. Although the difference was not statistically significant, an association between inferior staphyloma and dome-shaped macula could be suspected.

Thick choroid was suggested as one of the possible reasons for dome-shaped macula [1]. According to the report by Imamura and associates, the choroidal thickness was minimally thicker in eyes with dome-shaped macula when compared to that of eyes without dome-shaped macula [2]. The possible reason of the variation in elevation would be of scleral origin. Eyes with dome-shaped macula exhibited relatively thinner choroid than eyes without dome-shaped macula in the present study. We often observed extreme thinning of the choroid at the top of the bulge in eyes with dome-shaped macula. In the present study, eyes with dome-shaped macula had a relatively greater degree of myopia than eyes without dome-shaped macula. In our experience, there is a considerable regional variation in choroidal thickness around the subfoveal area in some of the eyes with degenerative myopia. For example, since eyes with degenerative myopia usually exhibit a very thin choroid, increase in the subfoveal choroidal thickness could be noted when a large choroidal vessel was located at subfoveal area. This variation may partially influence the different results within the studies. One recent study suggested an association between thin choroid and foveal serous retinal detachment in eyes with inferior staphyloma [18]. Further studies to verify the influence of dome-shaped macula on choroid would be of value.

In the present study, macular serous detachment (foveal detachment) without other macular complication was identified only in two eyes (5.7%). There was a considerable variation in the incidence of foveal detachment associated with dome-shaped macula in the studies. Although rates of 52.1 to 66.7% incidence were reported by several investigator groups [1, 13, 19], the incidence was only 1.8 to 10.2% in other studies [3, 5, 6, 10]. Liang et al. reported the incidence of 1.8% in their study [6]. The exact reason for this variation is not certain. One possible explanation is the different subjects’ characteristics, including ethnic differences among the studies. The incidence was relatively low in Asian subjects [3, 6, 10]. In addition, it has been reported that foveal detachment in dome-shaped macula may spontaneously resolve [20, 21]. It is possible that spontaneous resolution accounts for the differences in frequency observed. To date, none of the previous studies evaluated the true population-based prevalence of serous detachment in eyes with dome-shaped macula. An accurate prevalence rate requires confirmation based on further population-based studies.

In the study of Ellabban et al., the three-dimensional shape of the dome-shaped macula was evaluated [3], and classified into two subtypes. One was a band-shaped ridge within the staphyloma and the other was a dome-shaped convexity within the staphyloma. In the former subtype, a flat RPE line was usually observed in horizontal OCT scan images, whereas a convex configuration was observed on both vertical and horizontal OCT scan images in the latter subtype. In some patients, different subtypes of dome-shaped macula were observed in both eyes. In the present study, convex elevation of sclera was more frequently noted in vertical OCT scans. In approximately half of the patients, the elevation was noted in only vertical OCT scans, suggesting these patients may have had a ‘band-shaped ridge within the staphyloma’ subtype of dome-shaped macula.

The definition of dome-shaped macula has not yet been firmly established. Several different definitions of dome-shaped macula, including the convex elevation of macula [1, 2], an inward bulge in the macular retina, retinal pigment epithelium, and choroid [5], or more than 50 μm of elevation of the inward bulge [3, 10] have been used by different investigator groups. However, these definitions were merely arbitrary and the proper threshold value for diagnose dome-shaped macula has not been investigated. In the present study, we attempted to verify whether the cut-off value 50 μm is appropriate to diagnose dome-shaped macula. In our study, 85.7% of eyes with dome heights between 50 and 60 μm were diagnosed to have dome-shaped macula, whereas the proportion was only 61.5% in eyes with dome heights between 40 and 50 μm. Although the proportion was 100% in cases with dome heights of at least 60 μm, using this cut-off value may miss cases with smaller dome-shaped maculae. The optimal cut-off value obtained using the receiver operating characteristic curve was 48.5 μm and almost perfect agreement was obtained when compared to the 50-μm cut-off value used in our study, i.e., a value of 50 μm may be appropriate for diagnosis.

This study has several limitations. First, in addition to its retrospective nature, patients were included based on a computerized search using several specific terms. Thus, not all patients who might have met the inclusion criteria were included. Second, because this study was performed at a tertiary referrer center, it is possible that the characteristics of dome-shaped macula found in this study may be partially different from characteristics of dome-shaped macula in ordinary degenerative myopia.

## Conclusion

In conclusion, localized thickening and convex elevation of sclera were observed in eyes with dome-shaped macula, and its prevalence was about 40% among people with degenerative myopia. The presence of a high and large dome seemed to have a preventive effect on the development of myopic foveoschisis. Further studies may reveal a more detailed profile of dome-shaped macula and its clinical significance.

## Data Availability

The data used to support the findings of this study are available from the corresponding author upon request.

## Abbreviations

OCT: Optical Coherence Tomography
EDI: Enhenced depth imaging
BCVA: Best-corrected visual acuity
logMAR: Minimal angle of resolution
